# Seborrhoeic dermatitis and sebopsoriasis developing in patients on dupilumab: Two case reports

**DOI:** 10.1002/ccr3.2871

**Published:** 2020-05-09

**Authors:** Ali Al‐Janabi, Alexander M. Marsland

**Affiliations:** ^1^ The Dermatology Centre Salford Royal NHS Foundation Trust Manchester NIHR Biomedical Research Centre Manchester Academic Health Science Centre Salford UK; ^2^ Division of Musculoskeletal and Dermatological Sciences School of Biological Sciences Faculty of Biology, Medicine and Health The University of Manchester Salford UK

**Keywords:** adverse event, dupilumab, sebopsoriasis, seborrhoeic dermatitis

## Abstract

Cutaneous adverse events to dupilumab can be varied; this necessitates keeping a broad differential diagnosis to identify seemingly paradoxical reactions. It may be possible to treat the adverse event concurrently without stopping dupilumab.

## 1 INTRODUCTION

Dupilumab is a new biologic effective in the treatment of atopic dermatitis. New regional dermatoses occurring in patients on dupilumab, including rosacea, psoriasis, and contact dermatitis, have been reported and lead to treatment discontinuation in some patients. These are the first reports of seborrhoeic dermatitis and sebopsoriasis occurring on dupilumab.

Biologics have transformed the therapeutic landscape in Dermatology. Dupilumab is a biologic that has recently been shown to be effective in the treatment of atopic dermatitis.^1^ It targets interleukin (IL)‐4 and IL‐13; two cytokines key in Th2‐mediated immunity which drives atopic dermatitis. A meta‐analysis of randomized controlled trials identified conjunctivitis and injection site reaction as common adverse events in patients on dupilumab.^2^

Reporting adverse events in real‐world populations is a crucial step in improving treatment outcomes for patients. New regional dermatoses affecting the face are increasingly being recognized as an adverse event to dupilumab; these can be challenging to diagnose and can lead to discontinuation of treatment.^3^ Herein, we report two cases of patients presenting with new dermatoses while on dupilumab, and outline how these were managed successfully without discontinuation of their treatment.

## 2 CASE 1

A 72‐year‐old man started dupilumab for severe atopic dermatitis. Prior to starting dupilumab, his Eczema Area Severity Index (EASI) score was 28.2 despite treatment with ciclosporin. By week 4 of treatment, his EASI score reduced to 11.2 and by week 16 it was 9.6. He presented 4 months into dupilumab treatment with well‐defined, erythematous plaques with yellow scale affecting his scalp, eyebrows, and external auditory meatus. Dermatoscopic findings included dotted vessels and yellow scale, consistent with sebopsoriasis. This was treated with shampoos containing clobetasol propionate, salicylic acid 0.5%, and distilled coal tar 1%. He required intermittent treatment over an 8 month period to achieve sustained clearance and currently does not require any topical treatments. The patient had no prior history of psoriasis or seborrhoeic dermatitis. Dupilumab has continued and his atopic dermatitis remains well controlled.

## 3 CASE 2

A 28‐year‐old woman had severe atopic dermatitis despite multiple courses of ciclosporin. She was started on dupilumab, with a reduction in EASI score from 19.4 pretreatment to 3.7 at 16 weeks. She presented 7 months into dupilumab treatment with poorly defined, pink plaques affecting the hairline, inguinal folds, inframammary folds and natal. Dermatoscopic examination revealed yellow scale and dot vessels. She was diagnosed with seborrhoeic dermatitis and treated with tacrolimus 0.1% ointment and emollients with excellent effect described at follow‐up 3‐months later. She currently does not require maintenance topical treatment. As per case 1, this patient also had no prior history of psoriasis or seborrhoeic dermatitis.

## 4 DISCUSSION

The occurrence of new regional dermatoses developing in patients on dupilumab has recently been reported and includes rosacea, allergic contact dermatitis, psoriasiform eruptions, and eczematous rashes predominantly affecting the face.^2^, ^3^, ^4^ These cases represent the first reported of seborrhoeic dermatitis and sebopsoriasis occurring in patients taking dupilumab. These cases were diagnosed clinically by their distribution and morphology. Sebopsoriasis was favored in case 1 as the plaques were more well‐defined; yellow scale is a key feature of both conditions. However, a flare of atopic dermatitis cannot be excluded as a possible explanation. While the patients responded to topical treatments and the dupilumab was continued, severe or treatment‐resistant adverse events can lead to discontinuation of dupilumab, as reported in other cases.^5^, ^6^

The cause of these events is unknown. It is possible that cytokine imbalance introduced by Th2 inhibition could lead to the development of other disease phenotypes. In the psoriasis literature, atopic dermatitis has been reported as an adverse event to biologics, and it has been speculated that Th17 pathway inhibition allows upregulation of Th2‐mediated immunity.^7^ Conversely, there have been reports of psoriasiform eruptions, psoriasis, and inflammatory arthritis developing after initiating dupilumab, which could indicate upregulation of Th1/ Th17 cytokines.^3^, ^4^, ^8^, ^9^, ^10^ The etiology of seborrhoeic dermatitis is multifactorial, involving the interaction between intrinsic susceptibility, immunological defects, and fungal colonization.^11^ Introducing cytokine imbalances might influence the immune response to Malassezia, leading to the development of seborrhoeic dermatitis in susceptible patients. Similarly, Heibel *et al* have reported a case of rosacea developing on dupilumab therapy and speculated that Th2 pathway inhibition might promote Demodex proliferation.^6^

Reporting adverse events is crucial in developing awareness among clinicians and sharing treatment strategies. Furthermore, it can be the starting point of translational research which can further our understanding of the mechanisms of disease and adverse events in different patients, leading to personalization of therapy.

## CONFLICT OF INTEREST

None declared.

## AUTHOR CONTRIBUTIONS

AA: wrote the manuscript. AM: cared for the patients, and edited and revised the manuscript.
